# Dietary macro- and micro-nutrients intake adequacy at 6th and 12th month post-bariatric surgery

**DOI:** 10.1186/s12893-020-00880-y

**Published:** 2020-10-12

**Authors:** Maryam Ziadlou, Firoozeh Hosseini-Esfahani, Hassan Mozaffari Khosravi, Farhad Hosseinpanah, Maryam Barzin, Alireza Khalaj, Majid Valizadeh

**Affiliations:** 1 International Campus of Shahid Sadoughi University of Medical Sciences, Yazd, Iran; 2 Nutrition and Endocrine Research Center, Research Institute for Endocrine Sciences, Shahid Beheshti University of Medical Science, Tehran, Iran; 3 Department of Nutrition, School of Public Health, Shahid Sadoughi University of Medical Sciences, Yazd, Iran; 4 Obesity Research Center, Research Institute for Endocrine Science, Shahid Beheshti University of Medical Sciences, Tehran, IR Iran; 5 Obesity Treatment Center, Department of Surgery, Faculty of Medicine, Shahed University, Tehran, Iran

**Keywords:** Bariatric surgery, Sleeve gastrectomy, Roux-en-Y gastric bypass, Nutrient intake adequacy

## Abstract

**Background:**

Bariatric surgery (BS) is considered as an effective solution to control morbid obesity. Food restrictions resulting from the operation may decrease dietary nutrient intakes, particularly during the first year after BS. This study mainly aimed to assess the adequacy of dietary nutrient intakes at 6th and 12th month after BS.

**Method:**

Of the severely obese participants in the Tehran obesity treatment study in 2015–2016, 58 patients undergoing Roux-En-Y gastric bypass (*N* = 16) or sleeve gastrectomy (*N* = 42) were selected from Tehran Obesity Treatment Center. To assess the patients’ dietary intake, a three-day, 24-h dietary recall was obtained on three unscheduled days (two non-consecutive weekdays and one weekend day) at 6th and 12th month after BS. To evaluate the adequacy of nutrient intake, the patients’ intakes were compared to the current dietary reference intakes (DRIs), including estimated average requirements (EAR) or Adequate Intakes (AI).

**Results:**

The mean age of the participants (71% women) undergoing BS was 37 ± 8 years. Anthropometric parameters significantly decreased at the 12th month after BS. The percentage of energy from carbohydrate intake increased significantly between the 6th and 12th month after BS (*P* = 0.04). The mean ± SD of protein intake was lower than the recommended dosage with a dramatic decrease from 45 ± 30 to 31 ± 15 (g/day) between the two intervals (*P* = 0.001). The mean intake of saturated fatty acid (SFA) decreased dramatically (*P* < 0.001) from 6 to 12 month; however, the median intake of n3-polyunsaturated fatty acid (n3-PUFA) intake increased (*P* = 0.02). None of the participants showed nutrient intake adequacy in terms of biotin, fat soluble vitamins, pantothenic acid, potassium, and zinc. Moreover, less than 10% of the participants showed nutrient intake adequacy in terms of folate, magnesium, manganese, and calcium according to DRIs during the both intervals after BS.

**Conclusion:**

Bariatric surgery can reduce dietary intakes, which is more obvious 12 months after the surgery. Out of 21 micronutrients, nearly all could not met the EAR and were received < 50%, also had significant reduction from the 6th to12th month after surgery.

## Background

Bariatric Surgery (BS) has recently become of great interest in treating morbid obesity and obesity-related comorbidities such as metabolic syndrome, insulin resistance, and type 2 diabetes [1–5]. Roux-en-Y gastric bypass (RYGB) and sleeve gastrectomy (SG) are the most popular BS techniques worldwide [6, 7]. Despite the effectiveness of BS on losing weight and improving quality of life for morbid obese patients, nutritional deficiency, protein–calorie malnutrition, and muscle loss are of great concern since they may emerge due to changes in stomach volume, lack of nutrient intake, protein and solid foods intolerance, and malabsorption [8–16]. There is limited information about dietary intake evaluation after BS based on the global recommendation [12, 17–21]. Vanoh et al. showed that despite of supplementation the intake of vitamins E and D, also zinc and calcium did not meet reference nutrient intake (RNI) [19]. Furthermore, patients may poorly observe the recommendations regarding the regular consumption of supplements. a literature review of 15 studies on Nutritional deficiency after SG showed that, despite of supplementation the incidence of vitamin B1, B6, B12, and calcium deficiency is common due to both malabsorption and food restriction pattern [22] . In some other cases, standard supplements sometimes is not sufficient to prevent nutritional deficiency [23, 24]. The present study mainly aimed to evaluate dietary intake adequacy of wide range of macronutrients and micronutrients based on the recommended standards at 6th and 12th month after BS. Also, assessing the nutrient density and comparison of nutrient intake changes between two intervals was the next points.

## Methods

### Patient selection and anthropometric measurement

**Fig. 1:**
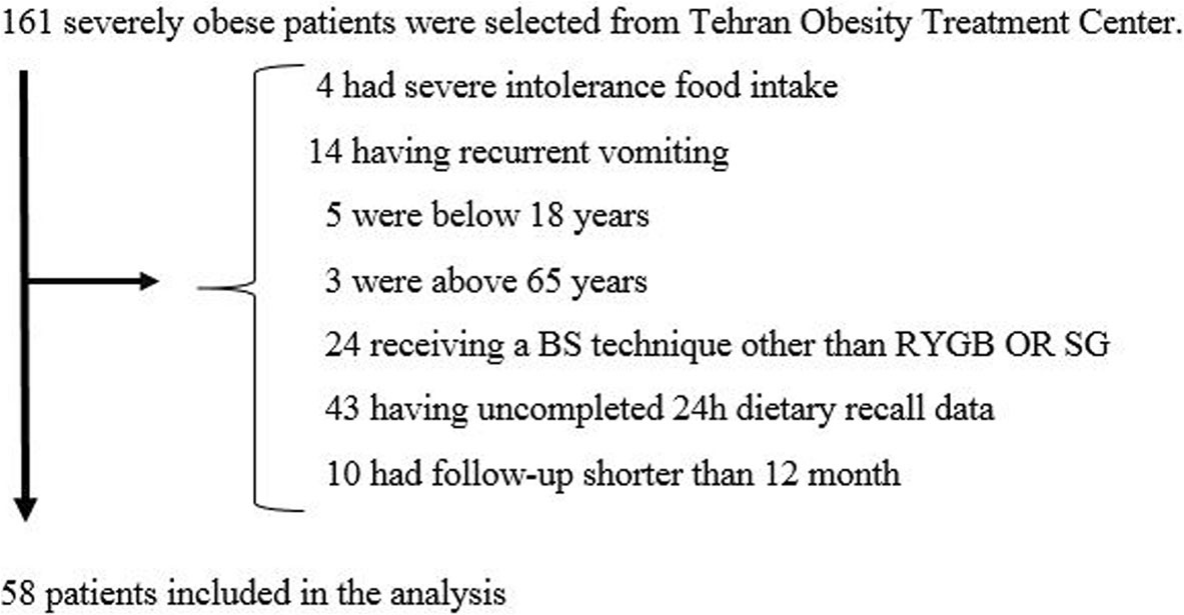
Patient selection criteria

This study was conducted in accordance with the framework of the Tehran obesity treatment study (TOTS), an ongoing single-institution prospective study initiated in March, 2013 [25]. In our retrospective study, 161 severely obese subjects with the body mass index (BMI) ≥40 kg/m2 or 40 < BMI ≥35 kg/m^2^ with at least one or more obesity-related co-morbidities undergoing BS during 2015 to 2016 were selected from the Tehran Obesity Treatment Center, 103 patients were not eligible to participate according to the exclusion criteria (Fig. 1). Fifty eight subjects undergoing either RYGB or SG were included in the present study. Sample size was calculated for at least 35 variables separately using sample size for comparing two dependent means, considering α = 0.05 and power 90%; the mean calculated samples was 56, only 7 variables were with calculated sample size of greater than 60 and 80% of variables had sample size lower than 60. Hence, the present sample size of this study seems sufficient to generalize to people undergoing surgery, also previous studies had similar sample sizes [10, 20, 26].

Anthropometric measurements were assessed before the surgery and at 6th and 12th month post-surgery in light clothes after overnight fasting. Moreover, BMI was calculated by dividing weight in kg by height in meters square, and the body composition analyzer *in Body 370* (Biospace, Seoul, Korea) was used to measure the body fat percentage and fat free mass (FFM). Further, waist circumference (WC) and hip circumference were measured to the nearest 0.2 cm with a flexible measuring tape, and height was measured to the nearest 0.1 cm using a wall-mounted stadiometer (Seca). The percentage of excess weight loss (%EWL) was calculated by standard formula: (pre-operative weight – post-operative weight) / (pre-operative weight –ideal weight) × 100 [15, 19] .

### Assessment of nutrient intake adequacy and nutrient density score

DRI was used to analyze energy as well as macronutrient and micronutrient intakes [27], and the estimated average requirement (EAR) cut-point was used to estimate the nutrient intake adequacy [27, 28]. Adequate Intakes (AI) was used for the micronutrients with no EAR value. Protein intake was assessed based on BS guideline recommendations [29–31]. The percentage distribution of the macronutrients with respect to total energy intake was assessed according to the acceptable macronutrient distribution ranges (AMDR). To assess the compliance of patients with dietary recommendations, dietary intakes were obtained using a three-day, 24-h dietary recall food questionnaire at the 6th and 12th month after BS (two non-consecutive weekdays and one weekend day). The questionnaire was filled out by a trained dietitian. The tables of the US Department of Agriculture [32] and the Iranian Food Composition [33] were used to convert crude data to grams, milligrams, or micrograms. The nutrient density score was also calculated to compare the rate of consuming healthy foods with lower energy density and higher nutrient-rich diets at the 6th and 12th month after the surgery. This score distinguishes nutrient-rich foods with high scores from high calories and poor-nutrients foods with low scores [34–36].

All the patients were scheduled for follow-up visits by a multidisciplinary team (ie, surgeon, obesity specialist, endocrinologist, and dietitian) at 10 days, and at 1, 3, 6, and 12 months after surgery. Our dietitian was responsible to consult the patients to follow healthy eating and dietary aspects of the regimen, including taking fresh fruits and vegetables, and elimination of sweetened beverages particularly for GB group to prevent dumping syndrome. Protein requirements was individualized according to gender, age, and weigh and should not be less than 60 g/day according to clinical practice guideline [29]. Daily Nutritional supplements were prescribed for all the patients according to ASMBS guideline, multivitamin Ecophan (Biorga) and Pharmaton (Boehringer-Ingelheim Inc. Ingelhem am Rhein, Germany) were prescribed in the month 6th and 12th after the surgery respectively. Calcicare as a calcium supplement was given to the GB group for a year, 1 tablet daily, containing 200 IU vitamin D, 400 mg Calcium, 100 mg magnesium and 4 mg Zinc per tablet [37].

### Statistical analysis

Analysis was performed using SPSS version 20 (SPSS Inc., Chicago, Illinois). The quantitative variables were summarized as mean ± standard deviation (SD) for normal variables and median and range for non-normal variables. Qualitative data were reported as frequency and percentage. The *Kolmogorov-Smirnov test* was used to determine data normality. Paired-t-test as a parametric test was used for the variables with normal distribution, to examine the difference of anthropometric variables and the dietary nutrient intakes between the 6th and 12th month after BS, and Wilcoxon signed rank test was used as nonparametric test in case of the data did not have normal distribution.

## Results

Of the 58 individuals participating in the study (RYGB (*N* = 16) and SG (*N* = 42)), 71% were female and 72% were married. The patients had a mean age of 37 ± 8 years. The mean weight, BMI, WC, and HC of the participants before the surgery were 120 ± 21 (kg), 44 ± 6 (kg/m2), 130 ± 16 (cm), and 133 ± 14 (cm), respectively.

### Anthropometric changes

Table 1 shows the characteristic and anthropometric parameters of the study participants before and after BS. The statistical analysis revealed a significant decrease in the mean weight, BMI, and WC in the first and second 6-month intervals for the post-surgery visit (*P* < 0.001). All the parameters decreased more rapidly during the first 6 months after BS. The percentage of body fat decreased significantly from 39 ± 8 to 34 ± 9% at the 6th and 12th month after BS, (*P* < 0.001). The percentage of EWL was > 61 ± 22 at the 6th month and increased to 76 ± 26 at the 12th month after BS (*P* < 0.001).

**Table 1:** Characteristic and anthropometric parameters of the study participants before and after BS

	Pre-operation *n* = 58	Post operation *n* = 58		*p*_value_
	Baseline	6th month	12th month	
Age	37 ± 8	–	–	
Gender (female)	71%	–	–	
Weight (kg)	120 ± 21	89 ± 17	83 ± 18	< 0.001
BMI (Kg/m^2^)	44 ± 6	33 ± 6	31 ± 6	< 0.001
WC (cm)	130 ± 16	106 ± 13	98 ± 13	< 0.001
HC (cm)	133 ± 14	115 ± 14	110 ± 10	< 0.001
FFM (%)	62 ± 11	53 ± 10	52 ± 10	< 0.100
Body Fat (%)	49 ± 3	39 ± 8	34 ± 9	< 0.001
Excess weight loss (%)	–	61 ± 22	76 ± 25	< 0.001

Data are presented as mean ± SD. *P*-value derived from paired t-test for numerical variables between the 6 and 12 month. *BMI* Body mass index, *WC* Waist circumference, *HC* Hip Circumference, *FFM* Fat-Free Mass

#### Macronutrient dietary intake changes and their adequacy

As shown in Table 2, the nutrient density had a significant decrease from 0.63 ± 0.20 to 0.44 ± 0.16 between the 6th and 12th month after BS (*p* < 0.001); however, the amount of carbohydrate intake increased significantly between two intervals (*P* = 0.04). Protein intake was 45 ± 30 g/d at the 6th month and decreased dramatically to 31 ± 15 (g/day) (*P* = 0.002) 1 year after the surgery.

**Table 2:** Daily dietary intake of energy and macronutrients and percent of individuals had adequacy intake at the 6th and 12th month post-Bariatric Surgery compared to dietary recommendations

Variables	Reference ^b^	Post-operation ^c^ (*n* = 58)		*P*-value	Adequacy ^f^	
		6 month	12 month		6 month	12 month
Total energy (kcal/day)	–	924 (3069)	1048 (2255)	0.057^d^	–	–
Nutrient Density (%) ^a^	–	0.63 ± 0.20	0.44 ± 0.16	< 0.001^e^	–	–
Carbohydrate (% of energy)	45–56	48 ± 12	53 ± 16	0.04 ^e^	100	100
Protein (g/d)	60 ^g^	45 ± 30	31 ± 15	0.002 ^e^	22.4	10.5
Total Fat (% of energy)	20–35	36 (47)	36 (65)	0.92 ^d^	44.8	34.5
SFA (% of energy)	< 10	15 ± 5	10 ± 5	0.02 ^e^	55.4	51.8
Linoleic acid (g/d)	11–17	4.2 (23)	4.0 (22)	0.997 ^d^	7.0	14
n3-PUFA (% of energy)	0.6–1.2	0.3 (1.0)	0.4 (5.8)	0.02 ^d^	17.5	28.1
n6-PUFA (% of energy)	5–10	5.5 (15)	5.6 (35)	0.651 ^d^	61.4	60.7
Fiber (g/d)	25–38	8.7 (31)	5.7(25)	0.001 ^d^	0	0

a.This score distinguishes nutrient-rich foods with high scores from foods with high calories and few nutrients with lower score

b.Dietary Reference Intake (DRI)

c. Data are presented as median (range) or mean ± SD according to normal and non-normal distribution respectively

d. P-value derived from Wilcoxon test for variables do not follow a normal distribution

e. *P*-value derived from paired t-test for normally distributed variables

f. The percent of individuals who had compliance with standard recommendation

g. The reference has considered based on BS Guide Lines protein recommendation

**Table 3:** Comparison of the dietary intakes and the percent of individuals with a dietary intake below the recommendations at the 6th and 12th months post-Bariatric Surgery

Variables	References ^a^	Post-operation ^b^ (*n* = 58)		*p*_value_	% of inadequacy ^e^	
***Vitamins***		6 month	12 month		6 month	12 month
Biotin (B7) _μg/d_	25–30	7.1 (29)	5.8 (30)	0.04 ^c^	100	98.2
Cobalamin (B12) _μg/d_	2.0	1.8 (3.8)	1.3(3.9)	0.001 ^c^	54.4	77.2
Folate (B9) _μg/d_	320–330	121(484)	102(496)	0.046 ^c^	93	96.5
Niacin (B3) _mg/d_	11–12	11.3 (36)	6.9 (30)	0.001 ^c^	47.4	71.9
Pantothenic acid (B5) _mg/d_	5	1.7 (4.3)	1.1 (4.5)	0.001 ^c^	100	100
Pyridoxine (B6) _mg/d_	1–1.4	0.9 ± 0.5	0.5 ± 0.4	< 0.001^d^	64.9	99.9
Riboflavin (B2) _mg/d_	0.9–1.0	0.63 (2.5)	0.52 (2.7)	0.033 ^c^	77.2	78.9
Thiamin (B1) _mg/d_	0.9–1.0	0.54 (1.6)	0.44 (1.4)	0.010 ^c^	86	91.2
Vitamin A _μg/d_	625–630	185 ± 144	110 ± 90	0.001^d^	99.2	100
Vitamin C _mg/d_	56–75	61.8 (193)	16.7 (110)	< 0.001^c^	50.4	99.9
Vitamin D _μg/d_	10	0.60 (2.6)	0.08 (1.8)	< 0.001^c^	100	100
Vitamin E _mg/d_	12	4.0 ± 3.8	4.6 ± 4.6	0.061^d^	100	86
Vitamin K _μg/d_	75–120	21 (158)	18 (123)	0.04 ^c^	100	100
***Minerals***						
Calcium (Ca) _mg/d_	800–1100	293 (985)	171 (799)	0.001 ^c^	93	98.2
Copper (Cu) _μg/d_	685–700	537(1737)	356 (1621)	0.002 ^c^	68.4	76.8
Iron (Fe) _mg/d_	6.0–8.1	5.2 (12)	4.6 (11)	0.014 ^c^	68.4	80.7
Magnesium (Mg) _mg/d_	255–340	115 (320)	89 (339)	0.013 ^c^	98.2	98.2
Manganese (Mn) _mg/d_	1.6–2.2	0.88 (3.1)	0.91 (4.2)	0.974 ^c^	96.5	91.2
Phosphorus (P) _mg/d_	580–1055	573 (1313)	441 (1088)	0.003 ^c^	50	71.4
Potassium (K) g/d	4.7	1.3 ± 0.6	0.9 ± 0.5	< 0.001^d^	100	100
Zinc (Zn) _mg/d_	6.8–9.4	3.9 (7.4)	3.2 (12.3)	0.009 ^c^	99.9	99.9

^a^EAR (An Estimated Average Requirement) is the average daily nutrient intake level estimated to meet the requirements of half of the healthy individuals in a group. EARs have not been established for vitamin K, pantothenic acid, biotin, manganese, and potassium, in this case AI (adequate intake) has been set. The cut point is adjusted for aged between 18 and 65 year; μg/d: microgram per day; mg/d: milligram per day

^b^Data are presented as median (range) or mean ± SD according to normal and non-normal distribution respectively

^c^P value derived from Wilcoxon test for variables do not follow a normal distribution

^d^P-value derived from paired t-test for normally distributed variables

^e^The percent of individuals who had inadequate intake

At the 6th month after BS, 22.4% of the individuals had adequate protein intake according to the BS recommendation [24, 25] but experienced a significant decrease in this regard 1 year after BS. Accordingly, only 10.5% of the individuals had protein intake adequacy and 50% of the individuals received below 29 g protein from foods.

The percentage of total dietary fat and n-6 PUFA intake revealed no significant difference at the both intervals, whereas SFA intake decreased dramatically between the 6th and 12th month after the surgery (*P* = 0.02) and 50% of individuals observed the recommendations and received < 10% of daily energy intake. Among the participants, the percentage of n3-PUFA from total energy intake increased significantly from 0.4 ± 0.2% to 0.8 ± 1.2% at the 6th and 12th month after the surgery (*P* = 0.02). Although, < 30% of the individuals had adequate intake at the both intervals. The median intake of fiber was 10.1 ± 8.7 g/d at the 6th month, which was significantly reduced to 6.9 ± 5.7 g/d a year after the surgery (*P* = 0.001). Fiber was the only macronutrient which was taken by none of the individuals according to the recommendations and 50% of population received < 9 g of fiber in both intervals (Table 2).

#### Dietary micronutrient intake changes and adequacy

As shown in Table 3, out of the 21 micronutrients, the dietary intake of 90% of nutrients had significant decreased between the 6th and 12th months after the surgery (*P* < 0.05). The dietary intakes of biotin (B_7_), fat soluble vitamins, niacin (B_3_), pantothenic acid (B_5_), pyridoxine (B_6_), thiamin (B_1_), as well as minerals such as calcium, copper, iron, potassium and phosphorus were extremely lower than the standard level at the both intervals. Of the participants undergoing BS, ≥98 showed inadequate nutrient intake in terms of biotin (B_7_), fat soluble vitamins, pantothenic acid (B_5_), potassium, and zinc (Zn), at 6th and 12th month after BS. Moreover, less than 10% of the individuals received calcium (Ca), folic acid (B9), magnesium (Mg), and manganese (Mn) according to the EAR or AI recommendations. Six months after the surgery, ≥65% of the participants had thiamin, riboflavin, pyridoxine, copper, and iron intakes below the recommended levels. This percentage increased up to > 90% for pyridoxine and thiamin and > 75% for copper, iron, and riboflavin 1 year after the surgery. Six months after BS, 50.4% of the patients had inadequate dietary intake for ascorbic acid, which increased to more than 99.9% 1 year after the surgery (*P* < 0.001). It suggests that less than 10% of the participants had dietary intake according EAR 1 year after BS.

## Discussion

This study evaluated the dietary intake adequacy of macronutrients and a wide range of micronutrients at the 6th and 12th month after BS and assessed the changes between two intervals. The findings showed that the percentages of carbohydrate and fat intake from energy were higher than the recommendations, and the percentage of carbohydrate intake increased significantly from 6th to 12th month after BS which is in contrast to the guideline claimed that to prevent weight regain in a short-term period, 40–45 and < 20% of calories should be provided from carbohydrates and fats 1 year after the surgery, respectively [29]. Also, the nutrient density score decreased dramatically between two intervals, it seems that the patients received high calorie poor-nutrients foods. Moreover, protein intake was observed to be lower than the recommended level at both intervals. According to previous studies, for patients undergoing BS, at least 60 g/day protein is adequate and 80–90 g/day protein is needed to prevent loss of lean body mass [29, 31]. Also, Heber et al., documented that patients should consume 30 g of protein in more than one meal per day to prevent bone and muscle insufficiencies [38]. In the present study, 77.6% of the participants had a protein intake below the standard (60 g/d) at month 6, and this value increased to 89.5% 1 year after BS. The results showed that 50% of population receive 39 and 29 g protein per day at the 6th and 12th month after surgery respectively. Our findings are in line with those of Mechanick, Andreu, and Moize et al. They showed that 61, 37, and 46% of the participants had protein intake < 60 g/day 1 year after BS [12, 39, 40]. In our study, the prevalence (%) of the patients with insufficient protein intake was approximately three times as large as the one reported by Andreu et al. 12 months after surgery [39]. The low protein intake adequacy may be the result of severe food restrictions caused by the small volume of the stomach and intolerance to protein-rich foods occurring 1 year after BS [14, 15, 41]. In our study, none of the participants could consume fiber according to recommendations. Low fiber intake is probably associated with low intake of vegetables and fruits due to the mechanical restriction imposed by the surgery. This finding is similar to the finding of Novais et al. who showed that participants’ dietary fiber intake was extremely low in accordance to the AI recommendations [18]. In the present study, the dietary intake of most micronutrients could not meet the EAR value at both intervals assessments and had significant decreased from the 6th to 12th month after BS. This is in contrast with data reported by Andrue et al. showed dietary intake increased gradually through a year post- BS [26]. The nutrients with severe low dietary intake were biotin, calcium, folate, pantothenic acid, fat soluble vitamins, vitamin C, potassium and magnesium which 50% of the population received ≤35% from the minimum EAR or AI 1 year after BS and more than 95% of patients could not meet the recommendations. Moreover, 77.2 and 80.7% of subjects showed inadequacy, In terms of cobalamin and iron intake respectively. The low dietary intake of iron and vitamin B12 was in line with previous studies [26, 42]. The high inadequacy might be related to the lower tolerance to meat, fish, dairy products, egg yolk, green leafy vegetables, nuts and seeds as the main source of these micronutrients. Similar to previous studies, the median intake of vitamin C was significantly decreased 1 year after surgery, and more than 99% of the participants had a vitamin C intake below EAR at the 12th month after BS. The high inadequacy of dietary vitamin C intake was in line with the findings of the previous studies [26, 42] and might be caused by the low quality of the diet with limited fruit and vegetable intake during the surgery follow-up period. As a matter of fact, after BS, patients need to be adjusted to the change in the gastric volume and tolerance for food, especially during the first year after the surgery. Wisnewsky et al., showed patients displayed a significant 2-fold decrease in food ingestion speed after BS [17]. Moreover, changes in eating behavior after BS like vomiting, difficult to swallowing due to texture, plugging (sense of food, particularly meat and bread becoming stuck in the upper digestive tract), reported as the barriers to food intake [43, 44]. Nutrient adequacy highly depended on food choices, food texture, and speed of food ingestion, volume and frequency of meal. The texture of foods provide patients to have better ingestion and digestion. Hence, following a proper and tolerable diet containing lean meat, poultry, fish, and legume in the blended form, also, pureed form of fruits and vegetables, fruit milk shake or fruit and yogurt smoothie with wheat germ/nut powder, soup prepared with bone broth and natural vegetable extracts can help the patients receive much micronutrients, fiber, protein, complex carbohydrates, and healthy sources of essential fatty acids naturally. This can promote a healthy short and long-term post-operative dietary pattern to prevent nutritional deficiency.

Furthermore, due to the low capacity of the stomach, the patients need to take high protein -low carbohydrate ready to use formula or drink enriched with vitamins and minerals specified for these groups to promote maximum adherence to their diet, patients undergoing BS are more susceptible to protein malnutrition [45–47], Schollenberger and Batar et al. showed that taking protein supplement can improve body composition by preventing FFM loss and enhancing loss of body fat [48, 49].

Recent studies have revealed that despite of supplementation, deficiency of cobalamin (vitamin B12), calcium, folate, fat soluble vitamins, thiamin and vitamin D is common after BS [22, 50–52]. Bariatric surgery are also associated with the risk for neurological complications (Wernicke encephalopathy and Korsakoff-syndrome) due to both shortage in dietary intake and non-compliance of vitamin supplementation [53, 54]. Researchers have claimed that supplement therapy may not be sufficient to prevent nutrient deficiency, the reasons are: prescribed supplements may not cover all target nutrients for individuals undergoing BS, moreover, recommended dosage may be insufficient, especially for those with malabsorptive operation (RYGB) [24], and patients may not regularly take supplement. Modi et al. showed that forgetting and difficulty swallowing supplement were the two main barriers identified for patients who had undergone BS [23]. Previous studies showed, numerous bioactive foods components (Apigenin, Allicin, Genistein, Luteolin, Lycopene, Myricetin, Quercetin, Resveratrol, Vitamins) can prevent cardiovascular disease and cancer [55–61]. These components are easily accessible in healthy foods mainly in fruits, vegetables, and whole grains [62]. Accordingly increasing the quality of their diet should be considered to promote individuals functional health. Registered dietitians are responsible to provide patients a practical dietetic recommendations according to the type of surgery and plan a proper diet based on the most common deficiencies according to the guideline like ASMBS to provide patient to meet nutrients intake from both foods and supplements [37]. Monitoring of patients in a shorter follow up period even monthly would be beneficial to provide them maximum adherence to the diet and supplements.

The main limitation of the present study was that there was insufficient data regarding the measurement of serum biochemical parameters before and after the surgery. Also, Lack of data in terms of preoperative food intake. A small sample size was another limitation of the study. Moreover, fewer Roux-En-Y patients (*N* = 16) than sleeve group (*N* = 42) was main barrier to do analyze between group. However, our study is outstanding for 1: assessing the dietary intake of a wide range of micronutrients and macronutrients according to the standards at the 6th and 12th month after BS and between two intervals. 2: evaluating nutrient density to distinguish healthy nutrient-rich foods from high-calorie and poor-nutrient foods.

## Conclusions

According to our findings, the dietary intake of protein, fiber, and nearly all the micronutrient especially biotin, calcium, folate, pantothenic acid, fat soluble vitamins, vitamin C, potassium and magnesium was significantly lower than EAR and significantly decreased between the 6th and 12month after BS. Eating a healthy, high quality tolerable diet is more important in the short and long time after the surgery because this operation has been done forever and it is expected the patients have adherence to both diet and supplements.

## Data Availability

The datasets used and analyzed in the present study are at the disposal of the corresponding Author and are available on reasonable request.

## Abbreviations

BS: Bariatric Surgery
DRIs: Dietary Reference Intakes
RYGB: Roux-en-Y Gastric Bypass
SG: Sleeve Gastrectomy
RDA: Recommended Dietary Allowance
RNI: Reference Nutrient Intake
TOTS: Tehran Obesity Treatment Study
BMI: Body Mass Index
FFM: Fat Free Mass
WC: Waist Circumference
HC: Hip Circumference
EWL: Excess Weight Loss
EAR: Estimated Average Requirement
AMDR: Acceptable Macronutrient Distribution Ranges
